# Reversal of rivaroxaban anticoagulant effect by prothrombin complex concentrates: which dose is sufficient to restore normal thrombin generation?

**DOI:** 10.1186/s12959-020-00228-9

**Published:** 2020-08-24

**Authors:** Lorine Giffard-Quillon, Helene Desmurs-Clavel, Claire Grange, Yohann Jourdy, Yesim Dargaud

**Affiliations:** 1 GEMMAT Groupe d’Etude Multidisciplinaire en Maladies Thrombotiques, Lyon, France; 2 Service de Medecine Interne, Medecine Vasculaire, Hopital Edouard Herriot, Lyon, France; 3 Service de Medecine Interne, Medecine Vasculaire, Centre Hospitalier Lyon Sud, Lyon, France; 4 Laboratoire d’Hematologie, Groupement Hospitalier Est, Hospices Civils de Lyon, Lyon, France; 5 Unite d’Hemostase Clinique, Hopital Cardiologique Louis Pradel, 28, avenue Doyen J. Lepine, F-69500 Bron, Lyon, France

## Abstract

Rivaroxaban has the most available data to support the use of prothrombin complex concentrates (PCC) as a reversal agent. However, PCC might increase the incidence of thrombotic events by shifting the haemostatic balance towards hypercoagulability. We assessed the in vitro efficacy and safety of three 4-factor PCCs for reversing rivaroxaban anticoagulant effect. Our in vitro finding indicates that 4-factor PCCs at the dose of 25 U.kg^− 1^ may be sufficient to reverse rivaroxaban anticoagulant effect.

Rivaroxaban is a direct oral activated factor X inhibitor. One of the main advantages of direct acting oral anticoagulants (DOAC) is the lower incidence of major bleeding complications, with 50% lower chances of intracranial haemorrhages compared with warfarin. However, DOAC may increase gastrointestinal bleeding and have been associated with heavy menstrual bleeding [1, 2]. Therefore, neutralization of the anticoagulant effect is a key step in the management of DOAC-related major bleeding complications. Andexanet-*α* is a catalytically inactive recombinant factor Xa protein that binds to both factor Xa inhibitors and native factor Xa (1:1 ratio), thus acting as competitive inhibitor. However, andexanet-*α* has been only conditionally approved by the European Medicines Agency because of the observed high incidence of thromboembolic events [3]. International guidelines recommend the administration of prothrombin complex concentrates (PCCs) in case of life-threatening bleeding caused by DOACs, in the absence of specific reversal agents [3, 4]. The initial dose of 50 U.kg^1^ is effective for achieving haemostasis and restoring thrombin generation. However, different groups have recently reported that PCCs at 25 U.kg^− 1^ provides effective anticoagulant reversal in patients with major bleeding complications associated with factor Xa inhibitors [5, 6]. Moreover, some experts suggested that activated PCC (APCC, FEIBA©, Takeda, Japan) could be an effective strategy to improve haemostasis in DOAC-related major bleeding events [7, 8]. Although clinical guidelines recommend PCCs for the treatment of DOAC-associated bleeding events [9], some authors suggested that PCCs may not be effective in this situation [10]. In the present study, we assessed the in vitro efficacy and safety of three 4-factor PCCs that contain the human coagulation factors II, VII, IX and X and are commercially available in France for reversing rivaroxaban anticoagulant effect.

After informed consent, blood samples of 40 patients who were treated with rivaroxaban (20 mg per day) after a venous thromboembolism were collected during a routine visit, at random times relative to the drug administration time. Rivaroxaban levels were measured using an amidolytic anti-Xa assay with drug-specific calibrators (HemosIL Liquid anti-Xa assay, Werfen, Le Pré-Saint Gervais, France) and a Werfen ACL top 750 analyser (Werfen, Le Pré-Saint Gervais, France). Rivaroxaban levels were high in four patients (200–300 ng.mL^− 1^), within the usual therapeutic range (31–200 ng.mL^− 1^) in twenty patients, and low in eleven patients (25–30 ng.mL^− 1^). Five plasma samples were excluded from the study because of undetectable rivaroxaban levels.

PCC reversal ability was tested using the thrombin generation assay (TGA) because the routine coagulation techniques to evaluate haemostasis in clinical laboratories are not sensitive enough to detect hypercoagulable and mild hypocoagulable states. TGA is a global haemostasis test that assesses the balance between procoagulant and anticoagulant proteins and might meet these requirements. Moreover, several groups previously reported that TGA can better reflect the bleeding or thrombosis risk of each individual patient compared with other routine coagulation assays based on activated partial thromboplastin time or prothrombin time [11]. For this assay, blood was collected in BD Vacutainer® tubes with 3.2% buffered sodium citrate solution and without corn trypsin inhibitor. For thrombin generation measurement, platelet-poor plasma samples were prepared as previously described for TGA [12], and spiked with three different PCCs available in France [Kanokad® (LFB, Courtaboeuf, France), Confidex® (CSL Behring, Marburg, Germany) and Octaplex®(Octapharma, Lachen, Switzerland)], to obtain different final concentrations (0–0.375 – 0.625 – 0.875 and 1.25 U.mL^− 1^) that correspond to the clinical doses of 0–15 – 25 – 35 and 50 U.kg^− 1^, respectively. Thrombin generation was measured using the Calibrated Automated Thrombin Generation Assay (Stago, Asnières, France) and the CAT PPP-reagent (Stago, Asnières, France), as previously described [12].

The presence of rivaroxaban in plasma modifies the appearance of the thrombin generation curve (i.e. “camel-back” shaped curve) [13], with delayed and reduced peak height and low endogenous thrombin potential (ETP). In our experimental conditions, the anti-Xa effect of rivaroxaban was more obvious on the thrombin peak height than on ETP (Fig. 1A). Therefore, the peak height was used as the TGA main parameter to assess PCC reversal effect. In the absence of PCCs, the peak height of samples from treated patients was 113 ± 53 nM (mean ± SD). Overall, rivaroxaban levels were negatively correlated with thrombin peak height (r = − 0.6241, CI 95% = − 0.83 – 0.26; *P* = 0.0019, Spearman correlation test). Peak height values were lower in patients with high rivaroxaban levels.

**Fig. 1:**
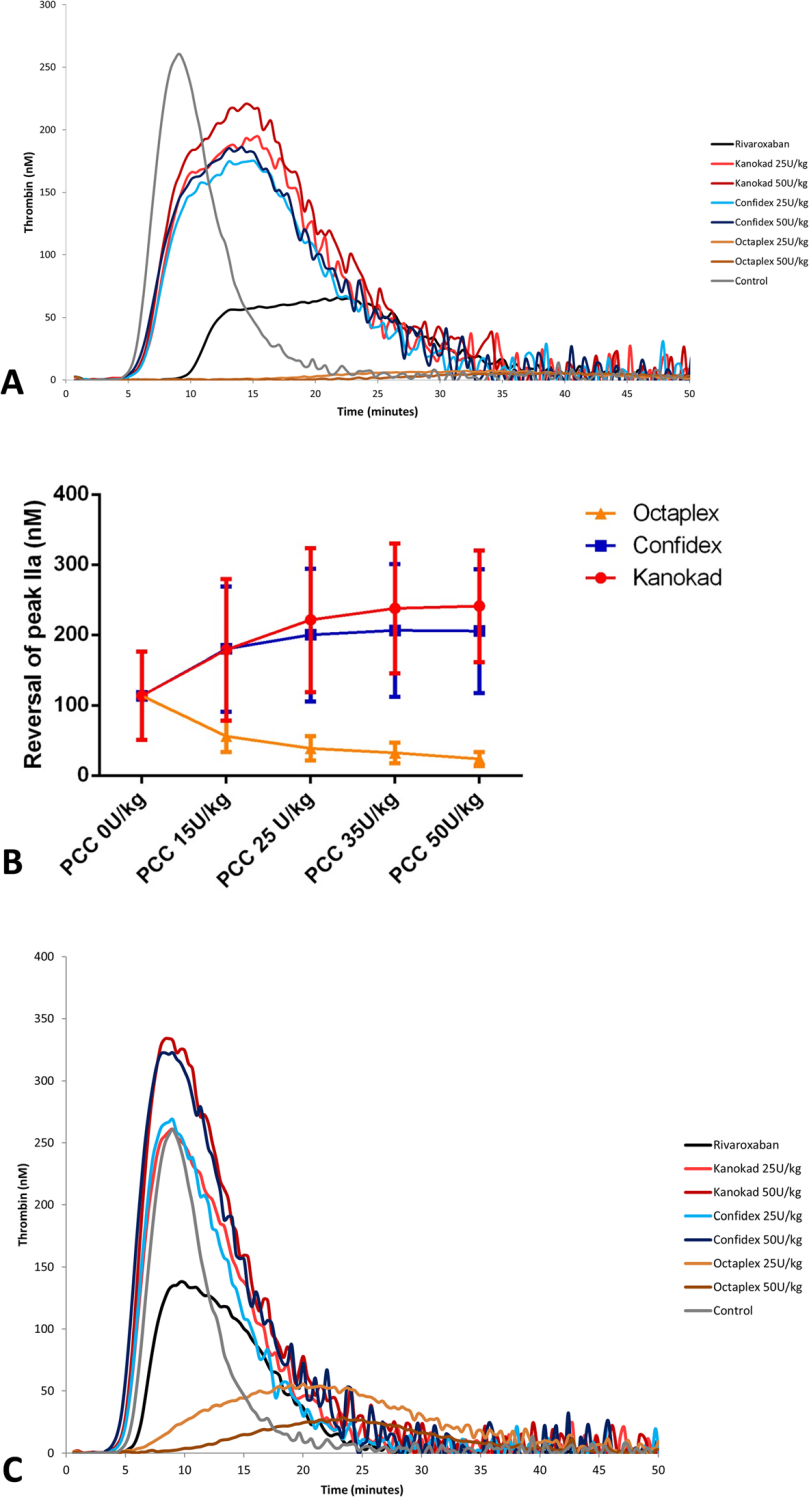
A. Typical “camel-back”-shaped thrombin generation curve obtained in patients treated with rivaroxaban (black curve). In this sample, rivaroxaban concentration was 89 ng/mL. In this sample, lag time was prolonged, and peak height and endogenous thrombin potential (ETP) were decreased compared with the normal control (grey). Lag time and peak height were restored after addition of the [4-]factor PCCs Kanokad® or Confidex® at the doses of 25 and 50 U/kg (0.625 and 1.25 U/mL respectively). Octaplex® did not have any effect in vitro, probably because of the high heparin content in this preparation. ETP was partially restored after PCC addition. Figure 1B. Dose-dependent changes of the in vitro thrombin generation peak in the presence of increasing concentrations of the indicated 4-factor PCCs. The four concentrations (0–15 – 25 – 35 and 50 U.kg^− 1^) correspond to the final concentrations of 0–0.375 – 0.625 – 0.875 and 1.25 U.mL^− 1^ in plasma samples containing rivaroxaban. Figure 1C. Thrombin generation curve of a sample with low rivaroxaban concentration (48 ng/mL). Before PCC addition, lag time was slightly prolonged, peak height was decreased, and ETP was normal compared with control. Lag time and peak height were restored after addition of the 4-factor PCCs Kanokad® and Confidex® at the dose of 25 U.kg^− 1^ (0.625 U/mL). In the rivaroxaban-containing sample, peak height and ETP were higher than in control after spiking with PCC 50 U.kg^− 1^ (1.25 U/mL)

In spiked samples, Kanokad® and Confidex® similarly increased thrombin peak height (*p* > 0.05; Mann Whitney test) at all tested concentrations. Thrombin peak height values and lag time were normalized with Kanokad® 25 U.kg^− 1^ (221 ± 92 nM) and Confidex® 25 U.kg^− 1^ (201 ± 94 nM) and remained in the normal range (Fig. 1A). Normal thrombin peak height values were previously determined in our laboratory in 100 healthy individuals without history of bleeding or thrombosis and who were not taking any medication that might interfere with coagulation. Thrombin generation measurement in these control samples in identical experimental conditions indicated that the normal peak height range was 280 ± 102 (mean ± 2SD) nM. The thrombin peak and ETP values obtained in patient samples spiked with Kanokad® and Confidex® (25 and 50 U.kg^− 1^) were not significantly different (*p* > 0.05; Mann Whitney U test) (Fig. 1B). Conversely, with lower dose of Kanokad® and Confidex® (15 U.kg^− 1^), thrombin peak and ETP values were significantly lower than in controls (*p* = 0.03 and 0.04 respectively; Mann Whitney U test). It is worth noting that in samples with low plasma rivaroxaban concentration, thrombin peak height reached values above the normal range after spiking with PCCs 50 U.kg^− 1^ (Fig. 1C). Quantification of heparin concentration in the PCC preparations showed that it was much higher in Octaplex® (0.3 anti-Xa units/mL) than in the other PCCs (< 0.1 anti-Xa units/mL). Consequently, thrombin generation peak and the area under the thrombin generation curve were inhibited in a dose-dependent manner in the presence of Octaplex®, and the in vitro results could not be reliably interpreted (Fig. 1).

PCCs are easy to obtain and can effectively reverse DOAC activity. They efficiently improve the coagulation parameters and decrease blood loss. However, dose recommendations are difficult due to lack of data, and the currently suggested dose for 4-factor PCCs is 25–50 U/kg [14]. On the basis of their potential thromboembolic risk and cost, it appears safe to use the minimum effective dose in patients with DOAC-related haemorrhages. Our in vitro results, obtained with two different 4-factor PCCs (Kanokad® and Confidex®) indicated that 25 and 50 U.kg^− 1^ led to an effective thrombin generation. However, they did not completely normalize all TGA parameters, particularly in samples with high concentrations of rivaroxaban. Indeed, lag time and velocity were not normalized in all samples with rivaroxaban levels higher than 200 ng/mL. This was expected because, unlike true “antidotes”, PCCs cannot inactivate FXa inhibitors.

The major limitation of our work is its pre-clinical in vitro design; no attempt was done to extend these results to clinical situations. Although PCC composition should meet precise criteria established by the European Pharmacopoeia, they differ in terms of inhibitor content (such as antithrombin), and concentration of heparin and of some coagulation factors. Moreover, batch-to-batch variations may be observed for the same brand. All these variations, associated with the in vivo pharmacokinetic characteristics of these complex molecules, make the extrapolation of in vitro results to clinical situations very difficult. Another limitation of our study was that blood samples were collected at random times and not at peak levels. Therefore, PCC effect might have been overestimated.

In agreement with our pre-clinical data, two recent clinical studies showed that in clinical situations, half of the patients treated with PCCs for urgent rivaroxaban or apixaban reversal received the dose of 25 U.kg^− 1^ (although 50 U.kg^− 1^ is the recommended dose), and had a good clinical outcome [15, 16]. Our in vitro finding and these clinical results indicate that 4-factor PCCs at the dose of 25 U.kg^− 1^ may be sufficient to reverse rivaroxaban anticoagulant effect. Moreover, the high peak height and ETP values observed in samples containing low concentration of rivaroxaban and PCCs 50 U.kg^− 1^ suggest that in some patients, high-dose PCCs might increase the risk of thrombosis. Although specific reversal agents for anti-Xa inhibitors are available, PCCs still have a role in centres without access to specific antidotes and also when the used anticoagulant agent is unknown. Large, prospective clinical studies are needed to determine the optimal dose of 4-factor PCCs for reversal of factor-Xa inhibitor activity.

## Data Availability

Yes
